# Aortic Valve Replacement Combined with Ascending Aortic Aneurysmectomy in a Patient with Sickle Cell Disease: a Case Report

**DOI:** 10.21470/1678-9741-2017-0185

**Published:** 2018

**Authors:** Lucas Molinari Veloso da Silveira, Ana Paula Tagliari, Ronaldo David da Costa, Cristiano Blaya Martins, Orlando Wender

**Affiliations:** 1 Hospital de Clínicas de Porto Alegre (HCPA), Universidade Federal do Rio Grande do Sul (UFRGS), Porto Alegre, RS, Brazil.

**Keywords:** Anemia, Sickle Cell, Cardiovascular Surgical Procedures, Extracorporeal Circulation, Aortic Disease/Surgery, Aortic Valve/Surgery

## Abstract

Sickle cell anemia is a haematological disorder characterized by multiple vaso-occlusive complications, resulting in a reduced life expectancy. These patients are exposed to several triggering factors for sickle cell crises when they are submitted to cardiovascular surgeries with extracorporeal circulation. Therefore, meticulous care and perioperative management are required. This paper reports a successful case of combined cardiovascular surgery - aortic valve replacement and ascending aortic aneurysmectomy - with no serious post-operative complications. In this report, we emphasize the peculiarities of perioperative care in patients with sickle cell anemia.

**Table:** 

Abbreviations, acronyms & symbols	
CI	= Confidence interval
Hb	= Hemoglobin
HbS	= Hemoglobin S
HR	= Hazard ratio
ROTEM	= Thromboelastometry
TEG	= Thrombelastography

## INTRODUCTION

Sickle cell anemia is an autosomal recessive hemoglobinopathy that affects approximately 5% of the population. It is characterized by chronic hemolytic anemia, recurrent painful vaso-occlusive crisis with progressive multisystemic damage, and reduced life expectancy^[1]^. However, improvements in its management and the increase in life expectancy observed in the last decades made possible an increase in the number of diagnosis of cardiovascular pathologies in patients with sickle cell anemia, and consequently cardiac surgical procedures are becoming more frequent in such patients.

Patients with sickle cell anemia submitted to cardiovascular surgeries with extracorporeal circulation are exposed to precipitating factors of crisis such as hypoxia, hypothermia, acidosis, and low flow states. This fact may lead to significant postoperative complications. For this reason, several precautions should be taken during transoperative period.

Due to the lack of evidence, this study aims to report a case of aortic valve replacement and ascending aortic aneurysmectomy, emphasizing aspects of the procedure and perioperative management. Currently, there is only one reported case of this surgery in such patients, which does not specify the management performed, making it difficult to establish a routine for complex procedures like this.

## CASE REPORT

**Fig. 1:**
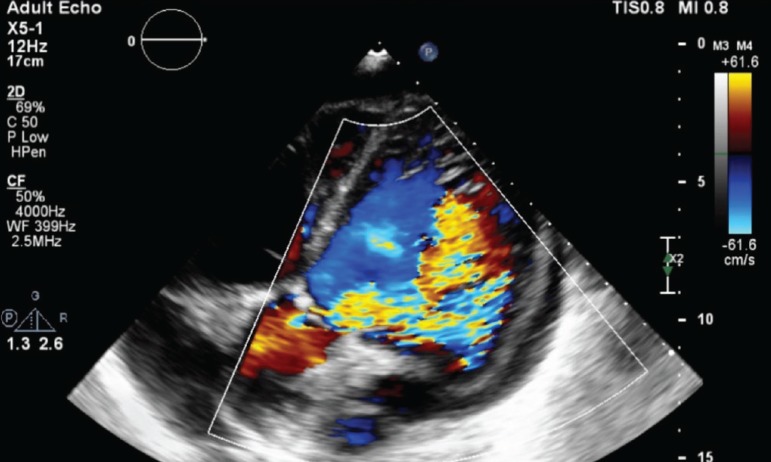
Transthoracic echocardiogram demonstrating severe aortic insufficiency.

**Fig. 2:**
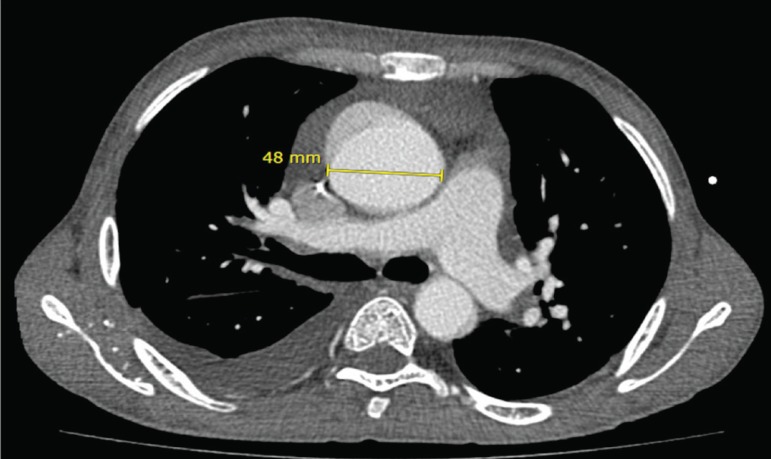
Contrast-enhanced computed tomography transaxial image demonstrating dilatation measuring 4.8 cm in the tubular portion of the ascending aorta.

A 30-year-old male patient diagnosed with SS sickle cell disease presented with sickle cell crisis and decompensated heart failure. Echocardiogram showed left ventricular dilatation (left ventricle end-diastolic diameter of 7.1 cm and end-systolic diameter of 5.1 cm), an ejection fraction of 53%, and a pulmonary arterial pressure of 70 mmHg, as well as a bicuspid aortic valve with severe insufficiency (Figure 1). At admission, the patient had hemoglobin (Hb) of 3 g/dL, hematocrit of 9%, and Hb electrophoresis with 89.3% of hemoglobin S (HbS). Angiotomography showed a 4.8 cm dilatation in the tubular portion of the ascending aorta (Figure 2). As a result of the multiple triggering factors for pulmonary hypertension, a right cardiac catheterization was performed, which demonstrated mean pulmonary artery pressure of 56 mmHg, mean capillary pressure of 30 mmHg, and pulmonary vascular resistance of 8.9 Wood units, characterizing a pulmonary hypertension by pre and post-capillary components.

Due to the challenges imposed by the case, a wide discussion was made by the institution's heart team. Considering the cardiovascular component's contribution in the symptomatology and that this prevented any reasonable quality of life, a surgical procedure was indicated.

In preoperative management, an exchange transfusion was planned aiming at hematocrit values > 25%, Hb > 10 g/dL, and HbS < 30%. However, simple blood transfusions were performed daily, which led to a hematocrit of 30.3%, Hb of 10 g/dL and 27.1% of HbS.

The patient underwent an aortic valve replacement and ascending aortic aneurysmectomy in October of 2016 at Hospital de Clínicas de Porto Alegre, a tertiary-level hospital. Anesthetic induction was performed with 12 mg of midazolam, 500 mcg of fentanyl, and 30 mg of rocuronium. Anesthesia was maintained with 0.7% isoflurane, midazolam, fentanyl, and rocuronium. Cardiac arrest was achieved by administering 20 ml/kg of Custodiol(r) hypothermic cardioplegia to the coronary ostia, according to the manufacturer's instructions, additional topical hypothermia was not used. During extracorporeal circulation, in order to avoid the occurrence of sickle cell crisis, the patient was maintained in normothermia (nasopharyngeal temperature above 34ºC) and hypoxia and acidosis were avoided, maintaining arterial oxygen saturation between 97.7% and 99.9%, and serum pH between 7.31 and 7.82. Resection of the native and ascending aorta was performed with subsequent implantation of a bovine pericardial prosthesis nº 25 and a supracoronary Dacron tube nº 26. Cardiopulmonary bypass and ischemic time were 95 and 85 minutes, respectively. There were no surgical or anesthetic complications. Cell saver was used throughout the procedure. Coagulation disorders were corrected based on activated clotting and thromboelastometry tests (ROTEM(r)).

In the immediate postoperative period, the patient presented hypovolemic shock and required an exploratory mediastinotomy, which didn't indicate the presence of active bleeding sites.

He presented a satisfactory postoperative evolution and he was discharged on the 18^th^ postoperative day, presenting complete symptoms resolution. In an outpatient follow-up, the patient showed significant symptomatic improvement and absence of major surgical complications.

## DISCUSSION

In the reported case, a patient with homozygous sickle cell anemia underwent a major cardiac surgery. Currently, aortic valve replacement surgery has a class I indication in patients with symptomatic severe aortic regurgitation^[2]^. Regarding aneurysmectomy, it is recommended in patients with a bicuspid aortic valve who will undergo aortic valve replacement, when the diameter of the ascending aorta > 4.5 cm^[3]^. Our patient's cardiovascular pathology was contemplated by both recommendations.

In this case, we chose to use a biological valve prosthesis based on various factors. First, patients with sickle cell anemia have a reduced life expectancy in relation to the general population. In addition, the use of a mechanical prosthesis would lead to the chronic use of anticoagulant medications, which are associated with hemorrhagic complications in anemic patients. Finally, we believe that the use of mechanical valve prosthesis should be avoided in these patients, since it could increase hemolysis and the risk of sickle cell crisis.

Another peculiarity of the reported case was the maintenance of normothermia, since hypothermia is known as a triggering factor for crisis. Usually, patients are maintained in mild hypothermia (> 32ºC) for myocardial protection and reduction of the metabolic and oxygen demand. However, hypothermia during cardiovascular surgeries in patients with sickle cell anemia is controversial. About half of the published case reports kept the patients normothermic. In those that opted for the use of hypothermic cardiopulmonary bypass, mild hypothermia (> 32ºC) was performed^[4]^. In a series of cases comparing hypothermia (various degrees) or not during extracorporeal circulation, there was no difference in complications incidence^[1]^. Considering that our patient had already presented several sickle cell crisis, we opted to minimize all the possible precipitators of a new crisis maintaining normothermia during the procedure.

We also emphasize that great importance has been given currently to the use of thrombelastography (TEG) and thromboelastometry (ROTEM) in patients undergoing cardiac surgery. For Kozek-Langenecker et al.^[5]^, hemostatic treatment guided by TEG or ROTEM reduces transfusion of both red blood cell, plasma, and platelet concentrate, providing a more restrictive blood product administration. The use of this technology allowed us to correct coagulation disorders more specifically and to focus on the real needs of our patient, avoiding transfusions that could be unnecessary and would lead to increased morbidity and mortality. Also, the use of cell-saver - a blood recovery system, which includes aspiration, filter wash, and then retransfusion of the blood to the patient - avoided homologous blood transfusions and improved hematocrit during procedure. The use of such technology is in consonance with current trends, since it is already proven that the number of transfused red blood cell units is an independent risk factor for clinical complications or death at 30 days (hazard ratio [HR] for each additional unit transfused = 1.2; 95% confidence interval [CI] = 1.1 - 1.4)^[6]^. In the majority of the cases reported in the literature, there is no objection to the use of cell-saver in patients with sickle cell disease. However, Sachithanandan et al.^[7]^ say that filtered and washed cell-saved blood is theoretically more prone to sickle-associated red cell deformation.

Finally, it was not necessary to perform an exchange transfusion, but simple sequential transfusions were performed. Collaborated for this fact the long period that the patient remained hospitalized before his surgery. In this way, it was possible that HbS levels of 27.1% were reached by various blood transfusions in sequence. Currently, there is no consensus regarding absolute HbS values for performing a surgical procedure safely. Nevertheless, it is proposed that it should be reduced to close to 30% in extensive surgical procedures^[8]^. Some authors recommend even lower values, such as < 10% for procedures that use extracorporeal circulation^[4]^. Regarding the fact that an exchange transfusion was not performed, there is currently no consensus about the transfusion method used to reach the preoperative goals of Hb and HbS^[4]^. However, most of the cases reported made use of preoperative exchange transfusion. Regardless of the form of transfusion used, we believe that it is necessary to reduce as much as possible the concentration of HbS.

## CONCLUSION

Patients with sickle cell disease are subject to many complications during cardiac surgeries and demand special measures during surgery to avoid those. However, little is known about the best perioperative management of these patients. This is the second reported case of a valve surgery combined with ascending aortic aneurysmectomy in a patient with sickle cell anemia, and we believe that this is the first case that describes the pre and transoperative management in details. This case demonstrates that it's possible to perform surgical correction of valvular pathologies and aortic aneurysms safely in patients with sickle cell anemia despite the limitations imposed by the disease.

**Table:** 

Authors' roles & responsibilities	
LMVS	Substantial contributions to the conception of this article, wrote and reviewed the paper, and approved the final version
APT	Substantial contributions to the conception of this article, wrote and reviewed the paper, and approved the final version
RDC	Substantial contributions to the conception of this article, wrote and reviewed the paper, and approved the final version
CBM	Substantial contributions to the conception of this article, wrote and reviewed the paper, and approved the final version
OW	Substantial contributions to the conception of this article, wrote and reviewed the paper, and approved the final version
